# AJP Invited Mini Review: Interstitial Cells and Arrhythmia

**DOI:** 10.1152/ajpcell.00882.2025

**Published:** 2026-03-02

**Authors:** Eva A. Rog-Zielinska, Jana Grune, Thorsten Kessler, Achim Lother, Peter Kohl

**Affiliations:** 1 Institute for Experimental Cardiovascular Medicine, University Heart Center and Faculty of Medicine, University of Freiburg, Freiburg, Germany; 2 Department of Cardiothoracic and Vascular Surgery, German Heart Center Berlin at Charité, Berlin, Germany; 3 Department of Cardiology, German Heart Centre, TUM University Hospital, Munich, Germany; 4 Department of Internal Medicine III – Cardiology and Angiology, Saarland University Medical Center, Homburg/Saar, Germany; 5 Institute of Experimental and Clinical Pharmacology and Toxicology, Faculty of Medicine, University of Freiburg, Germany; 6 Interdisciplinary Medical Intensive Care, Medical Center – University of Freiburg, Faculty of Medicine, University of Freiburg, Freiburg, Germany

**Keywords:** interstitium, arrhythmia, non-myocyte, hetero-cellular coupling, fibrosis

## Abstract

The electrophysiological relevance of interstitial non-myocytes for cardiac electrophysiology arises from their abundant direct and indirect interactions with cardiac myocytes. This mini-review defines the interstitium, explores biophysical and biochemical mechanisms of interactions between interstitial components and cardiac myocytes, illustrates consequences of these interactions for heart rhythm, and identifies targets for further research in this area.

## Graphical abstract

**Graphical abstract:**
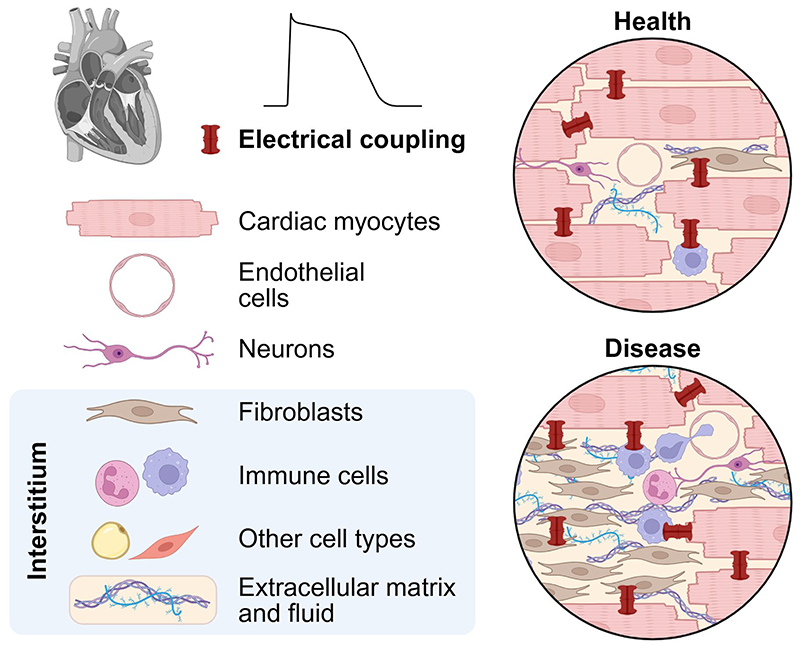

Interstitial non-myocytes, extracellular matrix, and extracellular fluid actively shape cardiac electrophysiology and contribute to arrhythmia susceptibility in heart disease through hetero-cellular electrical coupling, paracrine signalling, and pathological structural remodelling, with effects extending well beyond acting as a structural support and passive electrical insulator around cardiomyocytes. Image created with BioRender.

## Non-standard abbreviations used

AP: action potential(s)
AP_p_: pseudo-AP
CM: cardiomyocyte(s)
Cx: connexin
EC: endothelial cell(s)
ECF: extracellular fluid
ECM: extracellular matrix
FB: fibroblast
IC: immune cell(s)
MΦ: macrophage
NM: non-myocyte(s)
V_m_: trans-membrane voltage

## Introduction

The interstitium is a tissue constituent that encompasses the non-nerval and non-vascular cells, the extracellular matrix (ECM) and the extracellular fluid (ECF) in the space between those cells of an organ that underlie its main physiological function.

This definition builds on historic roots, following the 16^th^ century observation by Vesalius of “*a loose tissue in which nerves, blood vessels, and other fluids can flow*” (1). From the 18^th^ century onwards, this was referred to as the ‘interstitium’ (2). A century later, Virchow highlighted that the interstitium is functionally relevant, stating that “*interstitial tissue is both an essential site of, and a participant in, various pathological processes, as is the parenchyma itself*”, where parenchyma refers to the cells that underlie primary organ function (3). What these historic sources have in common is that they contrast the interstitium to the main working cells of a tissue, and that they exclude nerves and vessels – as those are part of separate ‘sprawling’ organ systems, which penetrate other organs of a body. More recently, this conceptual notion has been extended by the suggestion that the interstitium may be a sprawling organ system in its own right (4). While counterintuitive at first, the idea is conceptually intriguing, and based on the fact that a molecule within the ECF could travel to almost anywhere else in the body without ever leaving the interstitial space.

In this review, we will reflect upon the electrophysiological relevance of interstitial non-myocytes (NM) for heart rhythm that arises primarily from their biophysical interactions with cardiac myocytes (CM). Historically, and to some extent projecting through to the present day, cardiac electrical activity has been attributed solely to CM, while interstitial NM – together with the ECM – have been seen as ‘insulators’. However, we now know that NM not only form the majority of cells in the heart (5), but that that they can engage in direct electrical cross-talk with CM *in situ* in health and disease (6, 7).

### Configurations of Interstitial Cell Coupling to Cardiomyocytes

**Figure 1:**
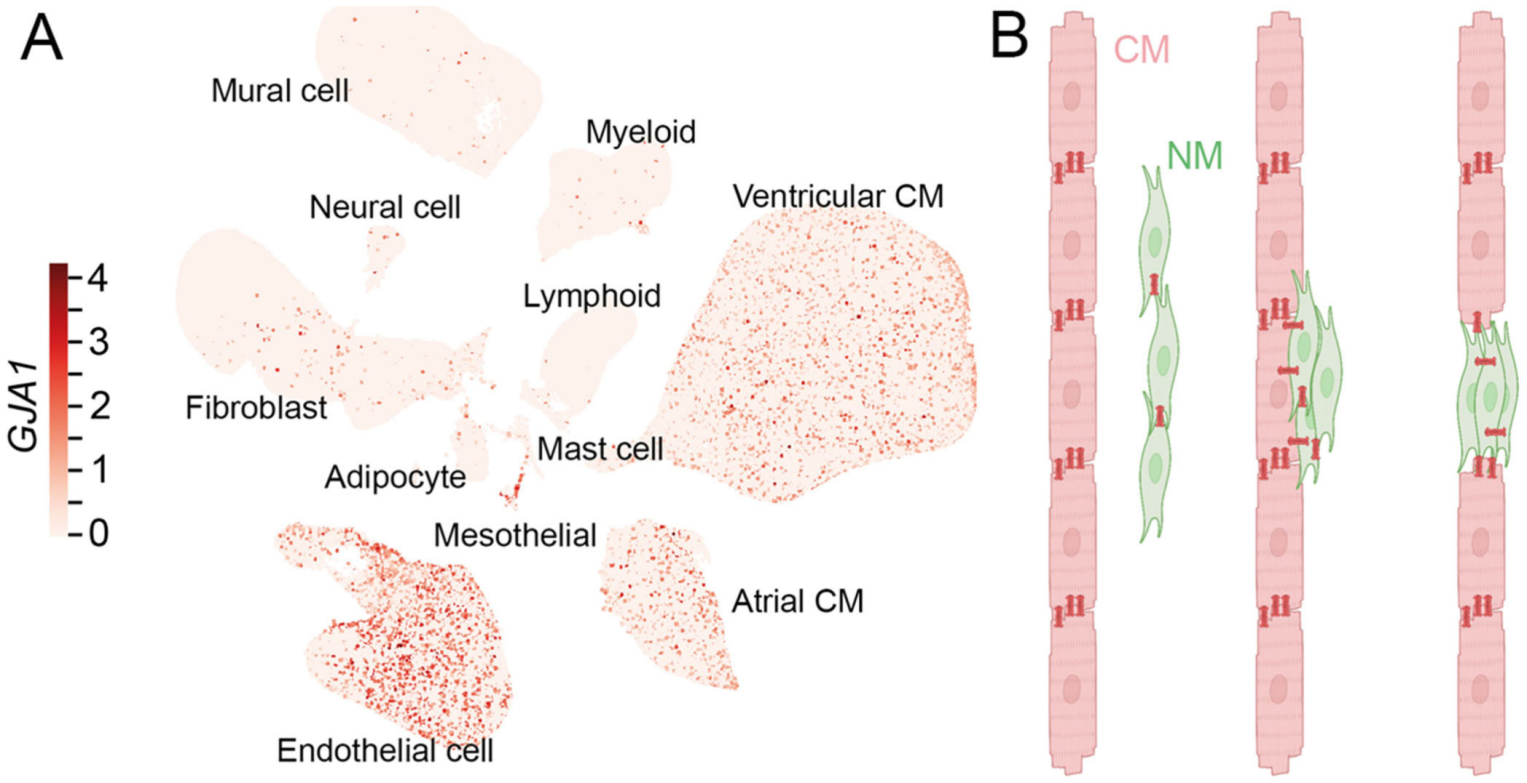
The hetero-cellular myocardium. **A:** Uniform manifold approximation and projection of cardiac cells showing expression of connexin 43 (*GJA1*) by cardiomyocytes (CM) and major non-myocyte (NM) populations, including endothelial cells, fibroblasts, adipocytes, mesothelial cells, and subsets of immune and mural cells in human heart (retrieved from https://www.heartcellatlas.org; (9)). Smaller NM populations, such as pericytes, melanocytes, or Schwann cells are not individually resolved. **B:** Possible configurations of hetero-cellular coupling (by connexins, dark red) of NM (green) to CM (light red). From left to right: 0-sided (no connection); 1-sided (electrical load); 2-sided (conductive bridge) connections. Icons from BioRender; see text for detail.

In contrast to CM, interstitial NM are electrically non-excitable. They do not express, *in situ*, the fast sodium channels that underlie the action potential (AP) upstroke in working myocardium, and they generally lack the types of calcium currents that are involved in pacemaker cell AP upstrokes. But since all cardiac NM for which identifiable data are available from recent large cellular sequencing databases (e.g. (8, 9)) express connexins (Cx), i.e. proteins that support electrical cell coupling (Fig. 1A), the ‘electrically passive’ cells of the cardiac interstitium may affect CM electrical behaviour.

Direct effects of interstitial cells on CM electrophysiology arise from three configurations of hetero-cellular coupling: zero-, single-, and double-sided coupling ((10); see Fig. 1B).

#### Zero-sided coupling

is functionally consistent with the classical view of cardiac electrophysiology, in which the cardiac interstitium is treated as an electrical insulator (Fig. 1B, left), while extending it by acknowledging that NM may be Cx-coupled with one another. Zero-sided coupling is likely to predominate in healthy working myocardium, as the electrophysiological consequences of NM–CM coupling appear subtle or negligible at the tissue level – even in studies reporting functional NM–CM coupling in remodelled myocardium (11, 12). That said, hetero-cellular coupling may be more prevalent and functionally relevant in pacemaker and conduction tissues, where NM–CM electrical coupling was first demonstrated (13, 14).

#### Single-sided coupling

**Figure 2:**
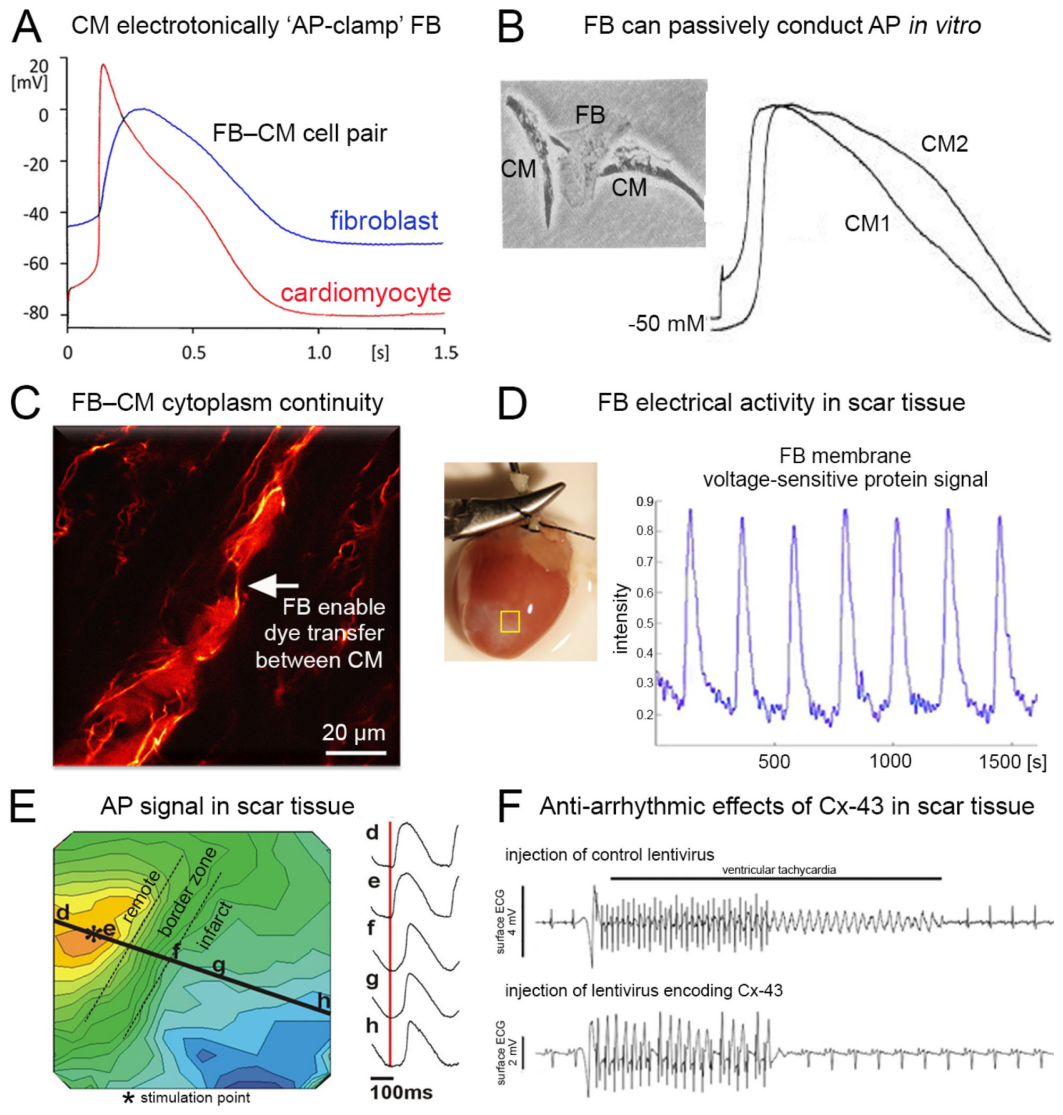
Key experimental observations, relevant for interstitial cell effects on cardiac electrophysiology, illustrating pseudo-action potential (AP_p_) generation and conduction *in vitro*, hetero-cellular coupling *in situ*, and the potential for therapeutic intervention *in vivo*. **A:** In freshly isolated neonatal rat CM and fibroblast (FB) co-cultures, FB coupled to CM demonstrate CM-like AP; these AP_p_ have a reduced amplitude and slowed upstroke (from (17)). **B:** In short-term culture (24 h), contracting neonatal rat CM, interconnected by a FB, show sequential AP generation (AP delay ~50 ms; from (19)). **C:** Lucifer yellow fluorescent dye spreads from a group of rabbit sino-atrial CM (large labelled cells at bottom-left) and FB (thin, brightly labelled cells) *via* a single FB (indicated by an arrow) to another CM, demonstrating cytosolic continuity between the different cell types in native tissue (from (13)). **D:** NM-targeted trans-membrane potential (V_m_)–sensitive fluorescent protein signals demonstrate AP-like electrical activity in murine cardiac scar tissue, confirming real-time CM–to–NM AP_p_ propagation (from (11)). **E:** Optical mapping of V_m_ illustrates epicardial activation from a stimulation site in peri-infarct myocardium (asterisk) towards and into a fully transmural post-ischaemic infarct (bottom-right) in a Langendorff-perfused rabbit heart, with slowed conduction (crowding of isochrones; each 2.3 ms apart) in the border zone, followed by acceleration inside the scar (left panel). AP-like V_m_ swings show slowed upstrokes and reduced amplitudes, compatible with passive AP_p_-transmission *via* NM (right panel; from (22)). **F:** Injection of lentivirus encoding GFP-tagged Cx43 into a ventricular scar (leading to Cx43 overexpression in interstitial cells) after transmural cryo-injury deceases the incidence of burst pacing-induced ventricular tachycardia in mice to 40%, compared to 80% in mice injected with control lentivirus encoding GFP only (from (23)). A,B,C,E: with permission; D: freely available online through the PNAS open access option; F: with permission under Creative Commons Attribution 4.0 International License.

means that NM attach to CM ‘in parallel’ (Fig. 1B, middle). Given their inherently less negative trans-membrane potential (V_m_), NM in this configuration depolarise resting CM, while also adding an electrical load that may affect AP dynamics, with potentially arrhythmogenic knock-on effects on CM excitability and refractoriness (as analysed in computational models (15)). Since cardiac NM have a comparatively high membrane resistance (GΩ-range) and a membrane capacitance that is about an order of magnitude smaller than that of CM (16), NM can be ‘AP-clamped’ by electrotonically connected CM (Fig. 2A (17)). Even though they are non-excitable, such NM will then passively display dampened (slowed dynamics, smaller amplitudes) V_m_ swings, which we shall call pseudo-AP (AP_p_) henceforth.

#### Double-sided connection

refers to NM that interlink CM ‘in series’ (Fig. 1B, right). This allows NM to form an electrotonic conduit for AP_p_ transmission between otherwise non-connected CM. Such electrotonic coupling has been observed *in vitro*, where it synchronises AP generation in CM that are not directly in contact with one another (18) *via* hetero-cellular gap junctions (Fig. 2B; (19)), and supports the passive spread of excitation over finite distances (up to the 10^-4^ m range in neonatal rat heart cultures (20)). While this behaviour had initially been assumed to be an artefact of cell culture models, where upregulation of Cx expression in NM may support electrical phenomena not seen *in vivo*, Lucifer yellow dye transfer (indicating direct cytoplasmic links between cells) has been reported between NM and CM in rabbit sino-atrial node (Fig. 2C (13)). Subsequent work confirmed dynamic electrical coupling of CM with interstitial NM, such as fibroblasts (FB) and tissue-resident macrophages (MΦ) in cardiac tissue regions that are rich in interstitial cells, e.g. after injury (Fig. 2D; (11, 21) or in pacemaking and conduction areas such as the atrio-ventricular node (14).

### Fibroblasts and Cardiac Electrophysiology

Cardiac FB are crucial for cardiac interstitial integrity. In disease, interstitial remodelling is frequently associated with fibrosis – an excessive accumulation of ECM, driven mainly by proliferating or recruited, activated FB (also referred to as myo-FB). The role of FB in shaping cardiac electrophysiology is multifaceted. Upon isolation and, in as far as this can be inferred, *in situ*, FB have a V_m_ of between -10 and -40 mV (16, 17, 24). In cell pairs, FB can modulate excitability and refractoriness of coupled CM by imposing a resistive load, that (i) causes depolarisation of resting CM, partially inactivating fast sodium currents that underlie the AP upstroke, while (ii) hastening early and delaying late AP repolarisation, which affects recovery of CM excitability. FB also alter CM AP morphology by forming a capacitive load, which further slows fast CM V_m_ changes, such as during the AP upstroke (12, 25, 26). All of these effects can create electrophysiological heterogeneities in cardiac tissue that may contribute to the formation of arrhythmogenic substrates.

#### Cx-based interactions

Electrotonic coupling between FB and CM has been attributed to Cx-containing gap junctions, which create electrical continuity between coupled cells. Hetero-cellular Cx43-based coupling has been demonstrated in native cardiac tissue (13, 27, 28). The effects of electrotonic coupling between FB and CM on whole-heart electrophysiology are non-linear, and depend on the resting V_m_, capacitance, and membrane resistance of coupled cells, as well as on CM AP shape, the extent of hetero-cellular coupling, and the rate of electrical excitation cycles. FB may form conduits for passive trans-scar conduction of AP_p_, for example after myocardial infarction (Fig. 2E (22)). As FB represent a low-pass filter for AP_p_ propagation (following fast V_m_ changes with a delay), trans-scar conduction may fail at elevated heart rates (29). Intriguingly, strategic steering of hetero-cellular Cx43-based coupling between FB and CM – either silencing (for example to reinforce the insulating effect of ablation lines (30)) or enhancing (for example to make small ventricular lesions electrically transparent, Fig. 2F; (23, 31)) – may represent attractive therapeutic strategies for treatment or prevention of cardiac arrhythmias. Given the comparatively short distances over which AP_p_ may be propagated purely passively, addition of electrically connected excitable cells (such as Cx43-transfected skeletal myoblasts (32)) can extend the maximum distance of trans-scar conduction by forming AP-generating repeater stations that recondition the transmitted signal (33).

#### Non-Cx-based interactions

A recent study reported that even after knocking-out Cx43, optogenetic depolarisation of FB in cardiac scars may still lead to electrical activation of the tissue (12), suggesting that other modes of coupling between FB and CM can be at play. Given the speed of responses, these could include ephaptic, direct cytosolic, or capacitive coupling. Ephaptic coupling occurs without direct cytoplasmic contact, *via* local electric fields that form when ion movements across membranes of two juxtaposed cells alter the ionic composition in diffusion-restricted intercellular cleft spaces (34). While the plausibility of ephaptic coupling between CM and FB has been demonstrated using computational models, the relevance of this mode of hetero-cellular interaction *in vivo* is uncertain. For CM–to–FB conduction, one needs to consider that FB are not excitable; their partial depolarisation would thus not trigger an AP or even an AP_p_, which would be needed to generate electrophysiologically relevant V_m_ swings that can support propagation of cardiac excitation to downstream CM. For NM–to–CM conduction, one should reflect on the fact that *de novo* formation of ephapses would require a focal dissolution of the basement membrane that encases the entire CM surface, with exception of their intercalated discs at sites of CM–CM contact (where, indeed, the relevance of ephaptic coupling as a contributor to electrical conduction, in particular during reduced gap junctional coupling, is now well accepted (35–37)). While trans-basement membrane interactions between FB and CM may be facilitated by FB-borne tunnelling nanotubes, as shown in native tissue (11), these nanotubes form minute punctate contacts with CM *in situ* (nanotube diameters are 10–200 nm (38)). This would limit the size of diffusion-restricted clefts that may form between the two cell membranes, and it is unclear whether these may be sufficient to drive ephaptic depolarisation of CM to reach the threshold for AP initiation. As the alternative, cardiac hetero-cellular coupling *via* direct cytosolic connection through tunnelling nanotubes, observed *in vitro* (39), has thus far not been confirmed in the heart *in situ*. Finally, capacitive coupling is based on transfer of electrical energy between two closely-approximated cell membrane areas without a direct connection or changes in extracellular ion composition. Similar to ephaptic interactions, capacitive coupling between FB and CM requires extended membrane areas that are in close juxtaposition. While theoretically possible, based on reported *in situ* cell geometries capacitive coupling is unlikely to be of physiological relevance, as generated currents would be too small to matter (40).

#### Paracrine interactions

In addition to direct electrophysiological effects on CM, FB affect cardiac electrophysiology *via* paracrine signalling. For example, transforming growth factor-β or interleukin-6 release by (myo-)FB can lead to changes in the expression of ion channels and to downregulation of Cx expression in CM (41, 42). In addition, FB can also have bulk structural effects on myocardial connectivity, where excessive FB and ECM aggregation can mechanically separate myocardial bundles, affecting electrical propagation and creating arrhythmogenic substrates (see also below), as part of the broad range of interstitial cell effects on cardiac electrophysiology.

## Immune Cells and Cardiac Electrophysiology

It is becoming increasingly evident that tissue-resident and circulating IC modulate stromal cell and organ function in healthy and diseased hearts, including effects on heart rhythm (43, 44). In steady state, resident MΦ are essential for cardiac tissue maintenance, such as by preserving CM energy metabolism via efferocytosis (clearing up of cellular debris, such as mitochondria shed from CM; (45–48)) and they contribute to the maintenance of capillary integrity (45, 49). Interstitial remodelling is accompanied by changes in number, phenotype, and spatial distribution of intra-myocardial IC, reflecting both expansion or resident and recruitment of circulating IC populations, at times with divergent effects on cardiac structure and function (50). Beyond immune functions, cardiac IC have emerged as modulators of myocardial electrophysiology through direct gap-junctional coupling, cytokine-mediated ion channel modulation, and immune-driven structural remodelling. The effects of IC on cardiac electrophysiology are complex, and depend on their type and origin (as an example, tissue-resident and bone marrow-derived MΦ have a different repertoire of stretch-activated ion channels (51) – highlighting the importance of considering cell sources when interpreting experimental findings), as well as the timing, e.g. post-injury.

### Resident MΦ modulate atrio-ventricular node conduction

MΦ have been reported to directly modulate CM electrical activity in the healthy heart. Resident and recruited MΦ in the heart have a V_m_ that – similar to FB – is half-way between CM resting and peak AP levels (typically between -10 and -40 mV (15), although more negative values (-60 to -90 mV) have been reported for subsets of cultured MΦ [(52)). In the atrio-ventricular node, resident MΦ have been shown to electrotonically couple to nodal CM *via* Cx43, potentially modulating atrio-ventricular conduction (14). The quantitative importance and general applicability of this mechanism across species and disease states is under investigation. Furthermore, MΦ have been shown to enhance the expression of sodium channels in CM, potentially altering their excitability and conduction properties (53). This modulation of ion channel expression suggests that MΦ play a role in the pathophysiology of cardiac electrical dysfunction, particularly in diseases where CM interactions with IC are dysregulated.

### Recruited MΦ drive chronic myocardial remodelling

IC can exert indirect effects on electrical function through contributions to fibrotic interstitial remodelling, particularly in the aftermath of cardiac injury. MΦ subtypes can adopt pro-fibrotic phenotypes, such as demonstrated for Trem2^+^ MΦ subsets, which have previously been associated with ECM deposition and fibrosis (54–57). This Trem2^+^ MΦ-driven fibrotic remodelling can disrupt normal electrical conduction and promote atrial fibrillation (58). Accordingly, silencing of Trem2^+^ MΦ has been shown to rescue sinus rhythm, highlighting potential therapeutic benefits of targeting IC to mitigate effects of interstitial remodelling and associated arrhythmia risk (59).

### Acute post-MI leukocyte dynamics contribute to arrhythmogenesis

Other IC subsets can play direct, arrhythmogenic roles early after myocardial infarction. In arrhythmia-prone mice, a substantial influx of neutrophils and monocytes into the heart occurs within the first few hours post-injury, well before excess ECM deposition would explain arrhythmogenesis. Depletion of neutrophils has been shown to prevent ventricular arrhythmias, while depletion of tissue-resident MΦ exacerbated arrhythmogenesis due to a loss of efferocytosis (60). This illustrates that IC actively contribute to onset and progression of ventricular arrhythmias. Moreover, IC-derived proteins such as lipocalin-2, generate reactive oxygen species, while the resistin-like molecule γ can target CM lipid membranes and induce CM death, directly promoting arrhythmogenesis (61). These findings highlight the complex interplay between interstitial IC and CM in arrhythmogenesis, which we are only beginning to understand.

## Endothelial Cells and Cardiac Electrophysiology

### Endothelial cells (EC)

are an essential part of blood and lymph vessels, providing a dense network of capillaries that surround every single CM and support supply of oxygen and nutrients to, and the removal of waste products, debris and excess extracellular fluid from the heart. In addition, endocardial EC cover the inner surface not only of coronary arteries and junctions to the great vessels, but also of heart chambers and valves. Although vascular EC do not belong to the interstitium, according to the definition given above, they modulate NM migration as they form a barrier between the blood and heart tissue, and they interact with CM and NM *via* direct coupling and paracrine signalling, thereby affecting cardiac (including interstitial) structure, mechanics, and electrophysiology in multiple ways.

### Ion channel-mediated effects

Capillary EC express a number of potassium, calcium, and non-selective ion channels (recently reviewed elsewhere (62)). EC are non-excitable and, hence, do not actively generate AP, but they can be connected to neighbouring CM, smooth muscle cells, and pericytes *via* gap junctions (Cx43 (63)). This network may be of autoregulatory relevance, as adenosine triphosphate (ATP) depletion in CM (for example during myocardial ischaemia) leads to the opening of ATP-sensitive potassium channels, which increases the capacity of CM to hyperpolarise electrically connected NM, such as EC, smooth muscle cells, and pericytes (63). This can cause vascular relaxation, increasing local blood flow and nutritional supply to CM, potentially countering arrhythmogenesis.

### Paracrine effects

Beyond these direct effects on smooth muscle and pericytes, EC also interact with CM and other NM *via* paracrine signalling that may alter cardiac electrophysiological properties. EC-derived nitric oxide, *via* cyclic guanosine monophosphate signalling, decreases intracellular calcium levels in CM, which may be anti-arrhythmogenic (64). In addition, co-culture experiments show that the presence of EC enhances maturation and electrical activity of iPSC-derived CM (65, 66), although the relevance of this for the mature adult heart is not certain. In conditions of EC dysfunction, associated with impaired balance of nitric oxide and reactive oxygen species production, increased expression of adhesion molecules facilitates leukocyte migration from the blood into the heart tissue (67), where they contribute to arrhythmogenesis (see previous section). In addition, EC can drive interstitial fibrosis *via* inflammatory and profibrotic signalling (68), aiding FB–to–myo-FB pheno-conversion, accumulation of ECM, and arrhythmogenesis. Whether endothelial–to–mesenchymal cell transition also contributes to tissue fibrosis (69, 70), and whether EC-driven interstitial remodelling is cause and/or consequence of arrhythmias, remains controversial (68).

More recently, lymphatic vessels have gained growing interest as regulators of cardiac ECF turnover (see below), ion homeostasis, and immune cell trafficking (71). Ablation of cardiac lymphatic vessels causes diastolic dysfunction and interstitial fibrosis (72), which may predispose to arrhythmia.

Taken together, the role of EC as a regulatory hub, controlling tissue inflammation and fibrosis, is increasingly well established, while direct EC contributions to electrophysiology and arrhythmogenesis are matter of ongoing investigation.

## Other Non-Myocytes and Cardiac Electrophysiology

### The heart contains a wealth of additional cell types

(8) that can express Cx (Fig. 1A). Whether this supports functionally relevant electrical coupling to CM *in situ* is not well explored for many cell types, including Schwann cells which express Cx43 and Cx32 (73), or adipocytes which express Cx43 (74) and whose electrical interaction with CM has been suggested to have arrhythmogenic effects in the heart (75). While this remains to be experimentally validated, arrhythmogenic effects of cardiac melanocytes, *via* Cx45 coupling to atrial CM, have been reported (76).

Independently of direct coupling, paracrine signalling, even of less abundant NM types, may alter cardiac electrophysiology. The secretome of epicardial adipocytes, for example, influences CM ion channel expression (77), and microRNAs in extracellular vesicles released from epicardial adipose tissue promote arrhythmogenic conduction slowing (78). Similarly, paracrine effects of mast cells can contribute to fibrosis in pressure-overloaded hearts and promote atrial arrhythmogenesis (79).

This illustrates that effects of NM on cardiac electrophysiology extend well beyond the ‘divide and conquer’ concept, originally thought to underly arrhythmogenic effects of fatty-fibrous infiltration (80, 81). Clearly, there remains much unchartered territory in the CM–NM interaction landscape.

#### Extracellular Matrix and Extracellular Fluid

The ECM consists of a variety of proteins and other macromolecules, such as glycoproteins and glycosaminoglycans. It represents a non-cellular, tissue-specific component of all organs including the heart (82). In the traditional understanding, the ECM was mainly regarded as a static scaffold (82). The composition of the ECM varies between organs. In the heart, characteristics of the ECM differ in various tissue regions, such as cardiac vasculature, atrial or ventricular myocardium, and valve tissue. In the myocardium, the ECM plays important roles in mechano-transduction, affecting cell shape and orientation, and aiding the integration of passive and active forces across the organ (83). ECM components furthermore play pivotal roles in embryonic development (84), and in the dynamic myocardial remodelling in the adult.

Physiological remodelling in development or during transient systemic challenges, such as elite athletic performance or pregnancy, is not typically associated with lasting changes in ECM presence and organisation (85, 86). In contrast, ageing and disease appear to involve altered interstitial NM crosstalk and tissue stiffening (87, 88). The perhaps best-known manifestation of pathological interstitial remodelling is fibrosis, commonly defined as an excess accumulation of ECM (a definition that may be in need of revision, as altered cross-linking of ECM components may profoundly affect tissue mechanics without manifesting itself in a detectable change in overall ECM presence (89)). Fibrosis can be diffuse/reactive ‘interstitial’ fibrosis (e.g. in atrial fibrillation or heart failure), patchy ‘infiltrative’ fibrosis (e.g. in systemic metabolic diseases such as amyloidosis), or focal ‘replacement’ fibrosis (e.g. after myocardial infarction). It is worth noting that various forms of fibrosis may coexist in the same heart (90). It is important to appreciate that – while fibrosis is not ‘the disease’ *per se* (and by no means necessarily its earliest manifestation (91)) – it poses substantial clinical challenges, as fibrotic regions can create spatial and functional heterogeneity in the myocardium, establishing substrates for conduction slowing, dispersion of repolarisation, and arrhythmogenesis (92, 93). Fibrosis can lead to mechanical inhomogeneities in the heart, which may be arrhythmogenic (94, 95), for example *via* mechanisms referred to as mechano-electric coupling (96), such as mediated by stretch-activated ion channels that are present in CM and interstitial NM (97).

Importantly, scars are not ‘dead tissue’ (98), and – while they are low in CM content – total cell density in mature post-infarct lesions is three-to-five times higher than that in healthy myocardium (as NM are substantially smaller than CM) (99). This opens up ample scope for NM and ECM interactions with CM. These interactions are often far reaching, and extending beyond simple one-way routes. One exciting facet of such interactions occurs through secretion of ECM-modulating enzymes by both NM and CM, in particular proteases (such as the ‘a disintegrin with thrombospondin motifs’ [ADAMTS] family). These enzymes dynamically modulate cardiac ECM composition and organisation, and dysregulated ADAMTS activity can create fibrotic, heterogeneous substrates that promote atrial and ventricular arrhythmias (100, 101). Another example of a complex interplay between CM, NM, and ECM is the recently described role of FB-secreted ‘Mothers against decapentaplegic homolog 7’ (Smad7), a downstream effector of transforming growth factor-β (TGF-β) signalling, in modulating not only ECM composition, but also myocardial inflammation through effects on MΦ activation (102), which may indirectly alter CM conduction and excitability. Similarly, ‘proprotein convertase subtilisin/kexin type 6’ (PCSK6), upregulated in CM under hypoxic conditions, has been shown to promote FB ECM deposition and local tissue stiffening (103). Finally, beyond purely biochemical signalling, cardiac mechanical activity can also drive ECM remodelling. As an example, a recent study highlighted that CM hypocontractility can induce ECM remodelling via a process that can be prevented through FB-specific knock-out of the mitogen-activated protein kinase p38 (104), This highlights the importance of a better understanding of interstitial cross-talk of both NM and ECM with CM in the context of the beating heart, as a foundation of novel therapeutic concepts.

The ECF is defined as ‘fluid outside of cells’; most of the ECF is interstitial fluid (in human about 11 L; another 3 L is in blood plasma), and this fills the spaces between cells in an organ and permits, among others, transport and exchange of molecules. Both the ECM and ECF are tightly connected, both spatially and functionally. The ECF in the heart is critical for hetero-cellular signalling, serving as an ‘incubator’ and ‘conduit’ for biochemical cues (e.g. for growth factors or matrikines (105)), determining trans-membrane ion gradients that drive cellular electrophysiology, and providing ‘lubrication’ for myocardial sheetlet sliding (106). Changes in ECF composition are one of the determinants of how myocardial ischaemia affects CM excitability, with potentially arrhythmogenic effects in particular of sudden reperfusion of previously ischaemic tissue. The associated swift normalisation of extracellular osmotic pressure may drive cell swelling and reperfusion injury (107), while swift recovery of CM excitability along acutely reperfused coronary arteries my give rise to unexpected excitation tunnelling through the affected myocardium and subsequent arrhythmogenesis (108). Recent findings further highlight the relevance of ECF in modulating cardiac function. As an example, extracellular vesicles in the ECF from neonatal hearts have been shown to enhance CM regenerative capacity (109), which may help preserve uniform conduction and prevent arrhythmogenic remodelling. Targeted modulation of cardiac ECF properties has remained difficult – but where explored, e.g. via stimulation of cardiac lymphatic vessel growth, it has been found to reduce edema after myocardial infarction, limiting fibrosis development, and potentially mitigating conduction slowing and decreasing the risk of arrhythmias (110).

Taken together, the rapidly emerging roles of ECM and ECF in the dynamic modulation of cardiac function highlight that studies into cardiac arrhythmia mechanisms should always take into account the highly integrated nature of interstitial signalling effects on cardiac electrophysiology (111).

## Relevance of the Interstitium for Heart Rhythm in Health and Disease

To conclude, interstitial constituents affect cardiac electrophysiology in ways that extend well beyond the classic concept of electrical insulation. During the three decades since first characterisation of Cx channels connecting pairs of freshly isolated cardiac FB and CM (16) and the two decades since confirmation of functional cytosolic coupling of CM and NM in native cardiac tissue (13), the notion of hetero-cellular electrical coupling has arrived in the general canon of cardiac electrophysiology.

Core electrophysiological effects of interstitial NM are a function primarily of inherent V_m_ differences and size (whole cell conductance, capacitance) of the interacting cells, as well as their coupling (both in terms of strength and configuration; Fig. 1). This makes it difficult to attribute specific arrhythmogenic effects to cell types *per se*, as different NM show substantial overlap in relevant properties (e.g. V_m_, cell size and capacitance, or ability to express Cx). At the same time, nominally ‘same’ cardiac NM may have subtypes with differing ion channel expression profiles and V_m_ (as described for cardiac MΦ; (15)). Matters get even more complex when additional ion conductances may be activated in interstitial NM as a consequence of their biophysical interaction with CM – for example if stretch-activated ion channels in NM were affected by neighbouring CM mechanics, or if voltage-dependent ion channels in NM were to behave differently as a result of cells being ‘AP-clamped’ by electrotonically connected CM. In addition, NM–NM coupling, too, is a relevant parameter, as – by ‘virtually enlarging’ the size of coupled NM – this will affect source–sink relations upon electrical cross-talk with CM. First indications of specific differences between cardiac interstitial NM show, for example, that FB form extensively interconnected networks in atria and ventricles, while MΦ populate healthy myocardium primarily as solitary cells (99). Therefore, many of the mechanisms underlying homo- and hetero-cellular coupling of different cardiac NM populations, their regulation and remodelling during aging and in disease, as well as the means for targeted steering remain underexplored.

Key open questions relate to:

(i) the biophysical effects of CM electrical and mechanical activity on cardiac NM, ECM, and ECF composition (some of which, such as ECF composition, are difficult to probe directly and dynamically);
(ii) the presence and regulation of hetero-cellular coupling of cardiac NM with one another and with CM in development, health, ageing, and disease (including dynamic changes in cardiac NM presence, distribution and properties, by proliferation, migration, trans-differentiation, and/or recruitment of circulating cells);
(iii) the physiological, pathophysiological, and therapeutic relevance of cardiac NM (for example in the context of ‘interstitium-sparing’ approaches, such as pulsed field ablation that aims to selectively eradicate CM during lesion generation);
(iv) the interplay of interstitial cells, ECM, and ECF signalling in maintaining cardiac function (i.e. exploring their autoregulatory relevance, rather than focussing on adverse remodelling).

For many of these questions, we are lacking suitable tools and techniques that would allow spatio-temporally accurate and cell-type specific or ECM/ECF-targeted intervention and observation. Optogenetic techniques have opened doors for new approaches to probing interstitial NM effects on cardiac electrophysiology with high spatial and temporal resolution (though these are not currently tailored to serve investigation of ECM and ECF), and real-time optical steering of cardiac hetero-cellular coupling is an obvious candidate for further development. Also, pharmacological targeting of specific interstitial components is highly desirable, as knock-out experiments may fail to provide definitive answers in complex autoregulatory systems (112). Computational modelling can help to link cell to tissue and organ level observations, including insight from advanced human stem-cell derived organoids and engineered heart tissue that help to explore novel therapeutic approaches targeting or employing the interstitium for patient benefit (113–115), and it will also be needed to project across species, to take basic research insight to clinical relevance (25).

Ultimately, the cardiac interstitium is a crucial part of the morphological make-up of the heart, and one whose full functional importance we are just beginning to glance.
